# Longitudinal change in lung function and subsequent risks of cardiovascular events: evidence from four prospective cohort studies

**DOI:** 10.1186/s12916-021-02023-3

**Published:** 2021-07-02

**Authors:** Yun-Jiu Cheng, Zhen-Guang Chen, Zhu-Yu Li, Wei-Yi Mei, Wen-Tao Bi, Dong-Ling Luo

**Affiliations:** 1 Department of Cardiology, The First Affiliated Hospital, Sun Yat-Sen University, Guangzhou, 510700 China; 2 NHC Key Laboratory of Assisted Circulation, Sun Yat-sen University, Guangzhou, China; 3 Department of Thoracic Surgery, The First Affiliated Hospital, Sun Yat-Sen University, Guangzhou, China; 4 Department of Obstetrics and Gynecology, The First Affiliated Hospital, Sun Yat-Sen University, Guangzhou, China; 5 Department of Cardiology, The Eighth Affiliated Hospital, Sun Yat-Sen University, Shenzhen, 518033 China

**Keywords:** Lung function, Change, Cardiovascular events, Coronary heart disease, Heart failure

## Abstract

**Background:**

Lung function is constantly changing over the life course. Although the relation of cross-sectional lung function measure and adverse outcomes has been reported, data on longitudinal change and subsequent cardiovascular (CV) events risks are scarce. Therefore, this study is to determine the association of longitudinal change in lung function and subsequent cardiovascular risks.

**Methods:**

This study analyzed the data from four prospective cohorts. Subjects with at least two lung function tests were included. We calculated the rate of forced respiratory volume in 1 s (FEV1) and forced vital capacity (FVC) decline for each subject and categorized them into quartiles. The primary outcome was CV events, defined as a composite of coronary heart disease (CHD), chronic heart failure (CHF), stroke, and any CV death. Cox proportional hazards regression and restricted cubic spline models were applied.

**Results:**

The final sample comprised 12,899 participants (mean age 48.58 years; 43.61% male). Following an average of 14.79 (10.69) years, 3950 CV events occurred. Compared with the highest FEV1 quartile (Q4), the multivariable HRs for the lowest (Q1), 2nd (Q2), and 3rd quartiles (Q3) were 1.33 (95%CI 1.19, 1.49), 1.30 (1.16, 1.46), and 1.07 (0.95, 1.21), respectively. Likewise, compared with the reference quartile (Q4), the group that experienced a faster decline in FVC had higher HRs for CV events (1.06 [95%CI 0.94–1.20] for Q3, 1.15 [1.02–1.30] for Q2, and 1.28 [1.14–1.44] for Q1). The association remained robust across a series of sensitivity analyses and nearly all subgroups but was more evident in subjects < 60 years.

**Conclusions:**

We observed a monotonic increase in risks of CV events with a faster decline in FEV1 and FVC. These findings emphasize the value of periodic evaluation of lung function and open new opportunities for disease prevention.

**Supplementary Information:**

The online version contains supplementary material available at 10.1186/s12916-021-02023-3.

## Background

The prevalence of impaired lung function is high, affecting approximately 10–20% of the general population [1, 2]. It has been believed that poor lung function may be a risk factor for a wide range of diseases [1, 3–12]. However, some researchers demonstrated that the importance of lung function as a disease indicator is driven by some other confounders [8, 13, 14]; the inverse relationship of respiratory function to disease outcome is ascribed to an underlying link between lung function and age [14].

It should be noted that lung function is constantly changing across the life course—growing from birth to teenager, plateauing in young adulthood, and declining with advancing age [15]; thus, single-time measurement of lung function with disease outcome was unable to determine the precise association. Thereby, the importance of a distinct course of lung function trajectories has been increasingly emphasized [15–18].

However, few studies have reported the association of longitudinal change in lung function and subsequent risks of cardiovascular (CV) events. Most of the existing studies are assessing the association between baseline lung function and cardiovascular risks [3, 10, 19]. Among studies with a longitudinal evaluation of lung function, studies are limited by short-term exposure duration [20], inadequate covariate adjustment [21, 22], or only restricted to chronic obstructive pulmonary disease (COPD) morbidity or mortality [23–25]. This may limit the ability to quantify the actual relations of longitudinal change in lung function to cardiovascular risk in the general population.

In view of the rather limited information currently available, we here performed a more comprehensive evaluation of lung function change over a 10-year observation and quantified the subsequent risks of CV events in four population-based cohort studies.

## Methods

### Study design and cohort

**Fig. 1:**
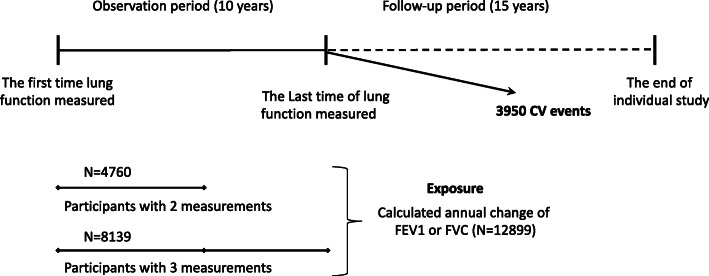
Description of the observation and follow-up periods of the study. CV, cardiovascular; FEV1, forced expiratory volume in one second; FVC, forced vital capacity

The present analysis was based on the data from 4 population-based cohort studies: (1) the CARDIA Study (The Coronary Artery Risk Development in Young Adults), (2) the CHS study (Cardiovascular Health Study), (3) the FHS Study (Framingham Heart Study), and (4) the FHS-OS Study (Framingham Offspring cohort). We obtained the cohort datasets from the NIH Biologic Specimen and Data Repository Information Coordinating Center (BioLINCC) [26, 27]. Details of the design of each study are reported in Additional file 1. Participants with at least two measurements of lung function tests within the first 10 years (observation period) were included. We excluded participants who were lost to follow-up or sustaining an event of interest during the observation period. After the last lung function measurement, 12,899 participants were included and followed up thereafter (follow-up period). The study flow is depicted in Fig. 1. The institutional review board approved the individual studies from the original cohorts, and all participants provided written informed consent in each study.

### Lung function assessment

Spirometry was performed with standardized equipment per protocol within the individual cohort. Details of measurements are provided in Additional file 1. FEV1 was assessed as the volume of gas exchange in the first second of expiration. Forced vital capacity (FVC) was assessed as the volume of gas forcefully exhaled after maximal inspiratory effort. We calculated the rate or slope for each subject to represent the annual change in FEV1 or FVC. The slope or rate of change was the coefficient computed from a simple linear regression model, in which each subject’s lung function (FEV1 or FVC) was considered as a dependent variable and the examination year as the independent one [22, 25]. The coefficient represents the direction and magnitude of lung function change. A positive value indicates an improvement in lung function while a negative one implies a decline. On the basis of previous publications [3, 10, 19], subjects were then categorized by quartiles of FEV1 or FVC decline over the 10-year observation period.

### Measurement of covariates

The covariates used for adjustment in this study were baseline sociodemographic variables (age, sex, race, education level, and marital status), past history (hypertension, diabetes mellitus, coronary heart disease, chronic heart failure, and COPD), health behaviors (smoking status, drinking status, physical activity, and body mass index), and biomarkers (fasting serum glucose, total cholesterol, high-density lipoprotein cholesterol, triglycerides, and low-density lipoprotein cholesterol). Seated or resting blood pressure was measured 3 times for each subject, and the average of the 2nd and 3rd measurements was used for the analysis. Height and body weight were measured in light clothing, and body mass index (BMI) was calculated by weight (kg) divided by the square of height (m^2^). A history of hypertension was defined by a ≥ 140 mmHg systolic blood pressure (BP) and ≥ 90 mmHg diastolic BP, self-reported hypertension, or using anti-hypertensive medications. A history of diabetes mellitus was defined by a ≥ 126 mg/dl fasting serum glucose, self-reported diabetes, or use of anti-diabetic medications. A history of coronary heart disease (CHD), chronic heart failure (CHF), and COPD was self-reported or being diagnosed by a physician. The level of physical activity was collected with self-report questionnaires and quantified using MET-min/week. Fasting serum glucose, total cholesterol, high-density (HDL-C) or low-density lipoprotein cholesterol (LDL-C), and triglycerides were measured following a standardized protocol. For covariates reported differently across the 4 cohorts, standardized categories were used to harmonize data across the cohorts.

### Ascertainment of the studied outcomes

Follow-up was started from the latest lung function measurement within this study. All participants were followed up to the event of interest, lost to follow-up, or until censoring at the end date of the individual cohort. All reported cases were systemically validated through medical review and adjudicated using each cohort’s specific protocol (Additional file 1). The primary outcome for our analysis was CV events, defined as a composite of CHD, CHF, stroke, and any CV death. CHD was defined as fatal or non-fatal myocardial infarction (MI), silent MI, or coronary revascularization, based on combinations of chest pain, electrocardiographic evidence, and change of cardiac biomarkers. The diagnosis of CHF required a constellation of symptoms or signs, along with a physician’s diagnosis of HF or an objective feature of pulmonary edema or ventricular dysfunction as described in previous publications [28, 29]. The secondary outcomes were CHD, CHF, and stroke.

### Data analysis

The differences across quartiles were assessed using analysis of variance for continuous variables and chi-squared tests for categorical variables. We reported age-adjusted event rates per 1000 person-years within each quartile. Non-adjusted or multivariable-adjusted Cox regression models were used to estimate the hazard ratios (HRs) and 95% confidence intervals (CI) of all studied outcomes by quartiles of lung function change, taking the highest quartile (Q4) as the reference group. Covariates adjusted in the multivariable model included age, sex, race, education level, marital status, history of hypertension, diabetes, CHD, CHF, COPD, smoking status, current alcoholic use, physical activity, body mass index, fasting serum glucose, total cholesterol, HDL-C, triglycerides, and LDL-C. The proportional hazards assumption was checked, and no evidence was suggestive of potential violation for any exposure-outcome associations.

Missing data were handled using full information maximum likelihood under the missing-at-random assumption in our main analysis. Missing values on baseline covariates using Markov chain Monte Carlo multiple imputation method before their inclusion in the fully adjusted models. The results from 10 multiple imputation cycles were combined together to draw a final output.

We further explored the association of annual lung function change (as a continuous variable) and different outcomes by graphing the restricted cubic spline curves. In addition, Kaplan-Meier functions were used to illustrate the event-free survival of individual outcomes across quartiles of lung function decline.

To mitigate potential bias, the following sensitivity analyses were performed: (1) excluding participants with missing data on baseline covariates and (2) restricting the analysis to participants with no known history of CHD, CHF, and COPD at baseline. Furthermore, subgroup analyses were conducted to evaluate whether the association differed by age (≥ 60 or < 60 years), sex (male or female), race (white or non-white), body mass index (normal weight, overweight, or obese), or smoking status (never, former, or current smokers). Potential effect modifications by these variables were examined by introducing a product term to the final model and determined by using likelihood ratio tests.

We did all these analyses with STATA/SE 15.1 (StataCorp, College Station, TX, USA). All statistical tests were two-sided with a significance threshold of 0.05.

## Results

### Baseline characteristics of the study population

The average age (SD) of the 12,899 participants was 48.58 (21.15) years. 43.61% (5625/12,899) were men, and 78.30% (10,100/12,899) were self-identified as white. Baseline characteristics, including sociodemographic information, health behaviors, and biomarkers, by quartiles of lung function decline, and different cohorts are summarized in Additional file 2: Table S1, Additional file 3: Table S2, and Additional file 4: Table S3. All of them had at least 2 measures of lung function, and 63% (8,139/12,899) were with 3 times.

During the first 10 years of observation, the most rapidly declining quartile (Q1) lost an average of 101 (93) ml of FEV1 and 113 (76) ml of FVC per year. Compared to those with no or slow decline in lung function (Q4), individuals in the group that experienced a faster decline (Q3, Q2, and Q1) were older, more likely to be white, less educated, and have higher body mass index. They were also more likely to be married, smoke, and drink alcohol.

### Decline in FEV1 and the studied outcomes

During an average follow-up of 14.79 (10.69) years, CV events were seen in 30.62% (3950/12,899) of the study participants. The age-adjusted incidence rates of CV events per 1000 person-years by quartiles of FEV1 decline (from Q1 to Q4) were 23.58 (95%CI 22.90, 24.27), 21.82 (1.17, 22.48), 19.40 (18.79, 20.03), and 19.42 (18.81, 20.05), respectively. Compared to subjects assigned to the highest quartile (Q4), the crude hazard ratios for the lowest (Q1), second (Q2), and third quartile (Q3) were 1.95 (95%CI 1.74, 2.19), 2.42 (2.16, 2.71), and 1.67 (1.48, 1.88), respectively. The associations were attenuated after adjustment for baseline sociodemographic variables, past history, health behaviors, and biomarkers but remained statistically significant in the lowest (Q1) (HR 1.33 [95%CI 1.19, 1.49]) and second lowest (Q2) quartiles (HR 1.30 [1.16,1.46]). The association patterns were identical in the analysis of secondary outcomes, the results of which are provided in Table 1.

**Table 1:** Hazard ratio (95% confidence intervals) of the studied outcomes with quartiles of FEV1 and FVC decline

Studied outcomes	Model	Q1	Q2	Q3	Q4
**FEV1 decline**					
Cardiovascular events	Non-adjusted	1.95 (1.74, 2.19)	2.42 (2.16, 2.71)	1.67 (1.48, 1.88)	Reference
	Adjusted	1.33 (1.19, 1.49)	1.30 (1.16, 1.46)	1.07 (0.95, 1.21)	Reference
Coronary heart disease	Non-adjusted	2.05 (1.72, 2.44)	2.05 (1.71, 2.45)	1.67 (1.39, 2.00)	Reference
	Adjusted	1.38 (1.16, 1.65)	1.08 (0.90, 1.29)	1.04 (0.86, 1.26)	Reference
Chronic heart failure	Non-adjusted	2.02 (1.72, 2.36)	2.53 (2.17, 2.96)	1.98 (1.69, 2.33)	Reference
	Adjusted	1.47 (1.25, 1.73)	1.39 (1.19, 1.63)	1.33 (1.13, 1.56)	Reference
Stroke	Non-adjusted	1.94 (1.60, 2.36)	2.49 (2.06, 3.01)	1.65 (1.35, 2.02)	Reference
	Adjusted	1.35 (1.11, 1.64)	1.34 (1.10, 1.62)	1.08 (0.88, 1.33)	Reference
**FVC decline**					
Cardiovascular events	Non-adjusted	2.72 (2.43, 3.04)	2.21 (1.97, 2.47)	1.47 (1.31, 1.66)	Reference
	Adjusted	1.28 (1.14, 1.44)	1.15 (1.02, 1.30)	1.06 (0.94, 1.20)	Reference
Coronary heart disease	Non-adjusted	2.89 (2.41, 3.46)	2.42 (2.01, 2.90)	1.74 (1.44, 2.10)	Reference
	Adjusted	1.45 (1.20, 1.75)	1.33 (1.10, 1.61)	1.25 (1.03, 1.51)	Reference
Chronic heart failure	Non-adjusted	3.22 (2.77, 3.75)	2.45 (2.10, 2.86)	1.43 (1.21, 1.69)	Reference
	Adjusted	1.35 (1.15, 1.58)	1.21 (1.03, 1.42)	1.03 (0.87, 1.22)	Reference
Stroke	Non-adjusted	2.67 (2.22, 3.22)	2.23 (1.85, 2.69)	1.25 (1.02, 1.53)	Reference
	Adjusted	1.28 (1.06, 1.56)	1.19 (0.98, 1.45)	0.94 (0.77, 1.16)	Reference

Adjusted model: adjusted for age, sex, race, education level, marital status, history of hypertension, diabetes, coronary heart disease, chronic heart failure, chronic obstructive pulmonary disease, smoking status, current alcoholic use, physical activity, body mass index, fasting serum glucose, total cholesterol, high-density lipoprotein cholesterol, triglycerides, and low-density lipoprotein cholesterol

*FEV1* forced expiratory volume in one second, *FVC* forced vital capacity

### Decline in FVC and the studied outcomes

Similarly, the age-adjusted incidence rates of CV events by quartiles of FVC decline (from Q1 to Q4) were 26.68 (95%CI 25.97, 27.42), 20.41 (19.77, 21.05), 19.01 (18.40, 19.63), and 18.15 (17.56, 18.76) per 1000 person-years, respectively. Compared with the reference group (Q4), the group that experienced a faster decline in FVC had significantly higher crude HRs for cardiovascular events (1.47 [95%CI 1.31,1.66] for Q3, 2.21 [1.97-2.47] for Q2, and 2.72 [2.43-3.04] for Q1). Following covariate adjustment, the HRs were attenuated to 1.06 (95%CI 0.94–1.20), 1.15 (1.02–1.30), and 1.28 (1.14–1.44) respectively for the third (Q3), second (Q2), and first (Q1) quartiles. A similar pattern of association was observed for the secondary outcomes (Table 1).

### Dose-response relationship and Kaplan-Meier survival analysis

**Fig. 2:**
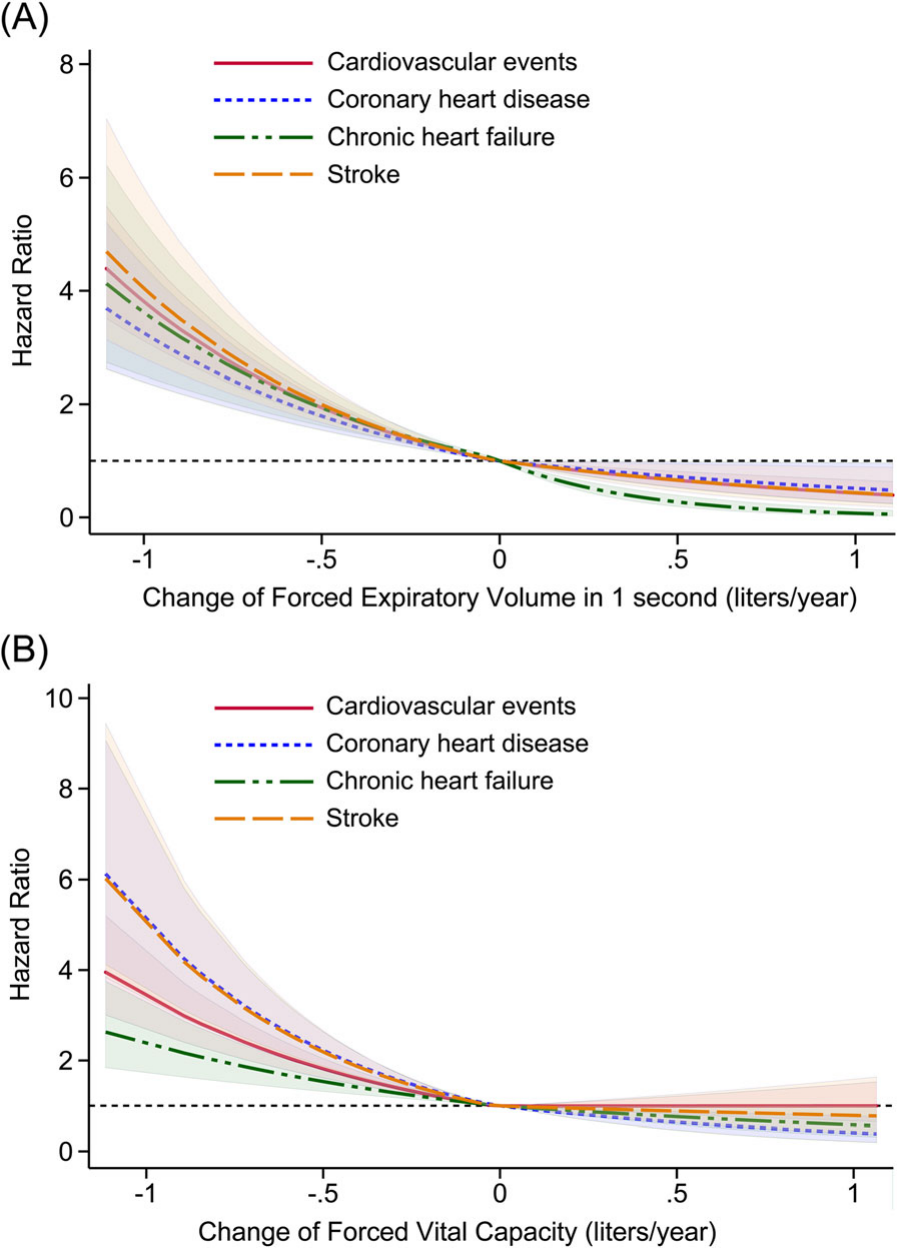
Dose-response relationship between the annual change in FEV1 (**A**) and FVC (**B**) and subsequent risks of the studied outcomes. The curves (solid or dotted lines) are plotted using restricted cubic splines and presented together with 95% confidence intervals (corresponding shaded area)

**Fig. 3:**
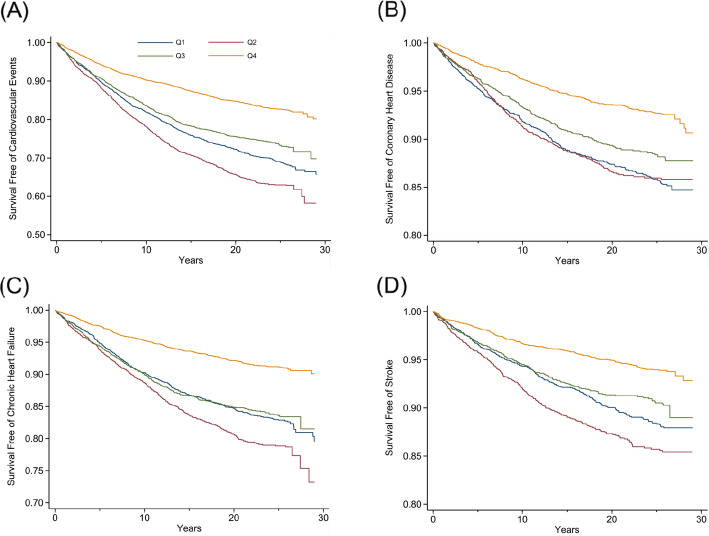
Kaplan-Meier event-free survival curves by quartiles of FEV1 decline. **A** Cardiovascular event-free survival curves by quartiles of FEV1 decline. **B** Coronary heart disease-free survival curves by quartiles of FEV1 decline. **C** Chronic heart failure-free survival curves by quartiles of FEV1 decline. **D** Stroke-free survival curves by quartiles of FEV1 decline

**Fig. 4:**
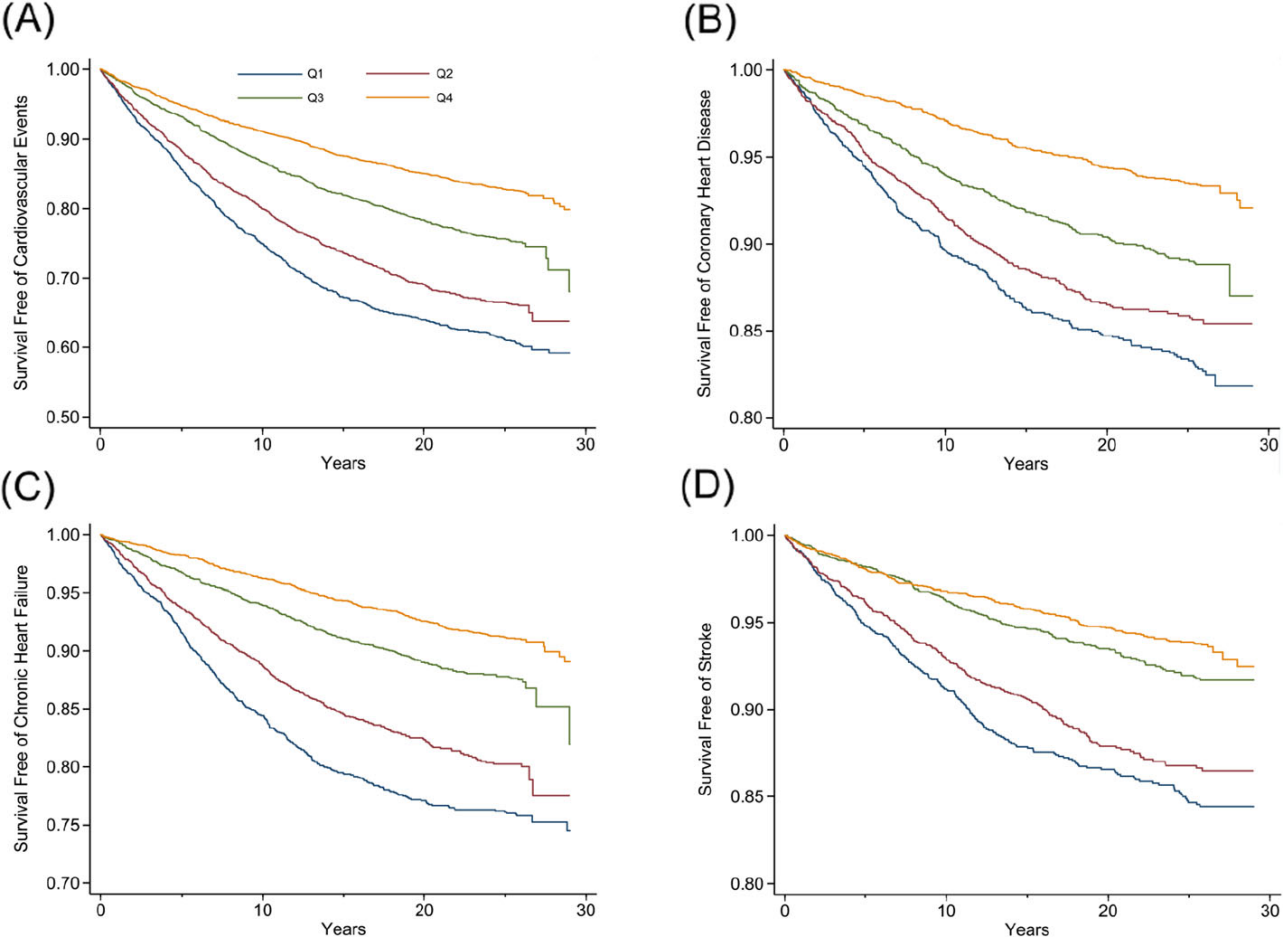
Kaplan-Meier event-free survival curves by quartiles of FVC decline. **A** Cardiovascular event-free survival curves by quartiles of FVC decline. **B** Coronary heart disease-free survival curves by quartiles of FVC decline. **C** Chronic heart failure-free survival curves by quartiles of FVC decline. **D** Stroke-free survival curves by quartiles of FVC decline

Similar associations were noted in the dose-response analysis, when modeling the longitudinal change in FEV1 and FVC as continuous variables. Figure 2A and B depict a monotonic increase in hazards of different outcomes with a faster decline in FEV1 and FVC. Furthermore, our unadjusted Kaplan-Meier results, grouped by quartiles of FEV1 (Fig. 3) and FVC (Fig. 4) decline, suggest a graded increased risk of CV events, CHD, CHF and stroke, with the lowest event-free survival observed in the lowest quartile (the most rapidly declining quartile).

### Stratification and sensitivity analysis

The findings were generally consistent in the stratification and sensitivity analyses. No interaction effects by sex, race, BMI, and smoking status were detected for the association between quartiles of FEV1 or FVC decline and the studied outcomes (Table 2 for the primary outcome and Additional file 5: Table S4, Additional file 6: Table S5 for the secondary outcomes). Age did significantly modify the relationship between lung function decline and the studied outcomes (P for interaction < 0.05), such that the associations with FEV1 or FVC decline were more evident in subjects younger than 60 years old. As for the sensitivity analyses, the associations were much the same when excluding participants with missing data on baseline covariates (Additional file 7: Table S6) or restricting the analysis to subjects with no known history of CHD, CHF, and COPD at baseline (Additional file 8: Table S7).

**Table 2:** Hazard ratios (95%CIs) of cardiovascular events with quartiles of FEV1 and FVC decline stratified by predefined subgroups

	Q1	Q2	Q3	Q4	*P* for interaction
**FEV1 decline**					
Age, years					
< 60	1.36 (1.10, 1.67)	1.25 (1.01, 1.56)	0.86 (0.68, 1.08)	Reference	< 0.001
≥ 60	1.23 (1.07, 1.42)	1.24 (1.08, 1.43)	1.12 (0.97, 1.30)	Reference	
Sex					
Women	1.20 (1.03, 1.41)	1.21 (1.04, 1.40)	0.98 (0.84, 1.14)	Reference	0.50
Men	1.49 (1.25, 1.77)	1.41 (1.18, 1.70)	1.17 (0.97, 1.42)	Reference	
Race					
Non-white	1.61 (1.21, 2.14)	1.07 (0.74, 1.54)	1.21 (0.86, 1.71)	Reference	0.95
White	1.30 (1.14, 1.47)	1.32 (1.17, 1.50)	1.06 (0.93, 1.21)	Reference	
Baseline BMI					
Normal	1.34 (1.11, 1.63)	1.14 (0.94, 1.38)	1.03 (0.84, 1.26)	Reference	0.85
Overweight	1.25 (1.04, 1.51)	1.37 (1.14, 1.65)	1.03 (0.85, 1.25)	Reference	
Obese	1.39 (1.10, 1.75)	1.37 (1.08, 1.74)	1.19 (0.93, 1.53)	Reference	
Smoking status					
Never	1.23 (1.02, 1.47)	1.19 (1.00, 1.42)	0.98 (0.81, 1.18)	Reference	0.33
Former	1.69 (1.29, 2.20)	1.65 (1.28, 2.12)	1.33 (1.03, 1.71)	Reference	
Current	1.28 (1.06, 1.54)	1.24 (1.01, 1.52)	1.00 (0.80, 1.24)	Reference	
**FVC decline**					
Age, years					
< 60	1.64 (1.31, 2.05)	1.34 (1.08, 1.67)	1.18 (0.96, 1.46)	Reference	< 0.001
≥ 60	1.13 (0.98, 1.30)	1.03 (0.90, 1.19)	0.98 (0.84, 1.14)	Reference	
Sex					
Women	1.25 (1.07, 1.47)	1.09 (0.94, 1.28)	1.01 (0.86, 1.18)	Reference	0.50
Men	1.32 (1.10, 1.58)	1.22 (1.01, 1.46)	1.09 (0.90, 1.31)	Reference	
Race					
Non-white	1.51 (1.10, 2.08)	1.15 (0.79, 1.67)	1.02 (0.70, 1.48)	Reference	0.95
White	1.26 (1.11, 1.43)	1.16 (1.03, 1.32)	1.06 (0.93, 1.21)	Reference	
Baseline BMI					
Normal	1.28 (1.06, 1.56)	1.10 (0.91, 1.33)	1.08 (0.89, 1.31)	Reference	0.85
Overweight	1.16 (0.96, 1.39)	1.02 (0.84, 1.23)	0.96 (0.79, 1.17)	Reference	
Obese	1.40 (1.09, 1.79)	1.50 (1.16, 1.93)	1.17 (0.90, 1.51)	Reference	
Smoking status					
Never	1.28 (1.06, 1.54)	1.20 (1.00, 1.44)	1.25 (1.04, 1.51)	Reference	0.33
Former	1.24 (0.94, 1.63)	1.15 (0.87, 1.53)	1.01 (0.75, 1.36)	Reference	
Current	1.36 (1.13, 1.63)	1.10 (0.90, 1.34)	0.87 (0.73, 1.08)	Reference	

Adjusted model: adjusted for age, sex, race, education level, marital status, history of hypertension, diabetes, coronary heart disease, heart failure, chronic obstructive pulmonary disease, smoking status, current alcoholic use, physical activity, body mass index, fasting serum glucose, total cholesterol, high-density lipoprotein cholesterol, triglycerides, and low-density lipoprotein cholesterol

*FEV1* forced expiratory volume in one second, *FVC* forced vital capacity

## Discussion

In this large population-based study, we showed a monotonic increase in risks of CV events, CHD, CHF, and stroke in subjects with a faster decline in lung function. The inverse association was independent of a series of socio-demographical factors and persisted after adjustment for traditional cardiovascular risk factors. Also, these findings were generally robust across sex, race, BMI, and smoking status but more evident in subjects younger than 60 years old.

### Comparisons with other studies

The importance of lung function decline is initially recognized in COPD populations [30, 31]. As demonstrated by Fletcher and some other researchers, COPD patients could experience different patterns of lung function trajectories, which were closely related to respiratory and all-cause mortality [16–18, 30, 31]. Since then, the impact of lung function decline on different disease outcomes is increasingly emphasized, even in the general population with normal or near-normal baseline lung function [20, 21, 23, 24, 32].

As expected, we found a graded relationship between quartiles of lung function decline and cardiovascular risks, which is consistent with the scarce existing data. It is been reported in the Baltimore Longitudinal Study of Aging (BLSA) that cardiac mortality generally increased with increasing quintiles of FEV1 [21]. Likewise, using data from the ARIC cohort, Odilson and colleagues reported a 14–34% increased risk of cardiovascular diseases in those with the fastest decline (120 ml/year) [20]. However, most of these investigations were based on a relatively short observation period of 3–4 years and failed to adjust factors associated with rapid lung function decline (i.e., alcohol consumption or education level) [22, 25, 33]. Our data complement these findings, making use of at least 2 measurements of lung function over a 10-year observation period and with a subsequent follow-up of 15 years. Additionally, the consistency of our findings in a dose-response analysis further confirmed the effect of lung function decline on the studied outcomes.

Quantitatively, our results appeared to differ from the existing data. It has been suggested that lung function declines with advancing age at approximately 23–32 ml/year for FEV1 and 14–30 ml/year for FVC in healthy, never-smoking subjects [15, 21]. However, in the current study, individuals in the second lowest quartile of FEV1 decline (Q2)—with an average decline of 37 ml per year—have already had at least 28% increased risks of cardiovascular events as compared with those in the reference group (Q4). This indicates that the threshold of lung function decline for increased risk of cardiovascular events is much lower than the perceived “normal value” reported in previous studies [15, 21].

### Effect modifications of some covariates

The increased risk of cardiovascular events in individuals with lung function decline was consistent across nearly all subgroups. But notably, this relationship was significantly modified by age. It surprised us that this association is more evident in younger individuals and only significant in the most rapidly declining quartile among the old. This is in line with the subgroup findings from an international-based cohort study, showing severe FEV1% impairment had the greatest effect on mortality of the subgroup younger than 50 years [7]. One proposed hypothesis for this observation is that older populations are at higher risk for cardiovascular events regardless of lung function, and thus, lung function may not provide additional prognostic information in this setting. Smoking status is often considered as an effect modifier when evaluating lung function; however, no significant interaction was seen in our analysis. Although a similar finding has been reported by Anthonisen et. al, in which smoking habit did not significantly influence the association of FEV1 decline and mortality risk [25], further research is needed.

### Potential mechanism and clinical implications

The reason for the association between longitudinal change in lung function and subsequent risk of cardiovascular events is not fully explained, but a number of mechanisms have been suggested. One is that reduced vital capacity is an indicator of biologic aging [8] and frequently shared similar risk factors with cardiovascular diseases [7]. However, the association persisted after adjustment of the above risk factors, implying that there is a real interplay between lung function decline and cardiovascular events. It is also been proposed that rapid lung function decline is associated with chronic inflammation [6, 34], which could induce substantial remodeling of the airway or respiratory structure [15, 35]. This would subsequently lead to ventilation/perfusion mismatch, causing progressive impairment of oxygen delivery and end-organ ischemia [12, 36, 37]. On the other hand, owing to lung function impairment, the ability of capturing and eliminating external toxic agents through the lungs would be compromised [6, 38] and the exposure insults could directly damage the heart.

It is clear from our study that accelerated lung function decline contributes significantly to cardiovascular diseases. Given the observational nature of this study, we could not confirm the cause-effect relationship between lung function decline and cardiovascular risks. However, following the Bradford Hill Criteria [39], the temporal relationship of a preceding exposure and subsequent events, the consistent findings across a series of analyses, and the evidence of a dose-response increase in risks gave further support to the causality. Generally, the current findings help to identify the high-risk populations and open new opportunities for prevention and early intervention. As previously presented in COPD populations, smoking cessation is able to alter the natural course of lung function decline [18]. Furthermore, avoiding dust and endotoxin exposure and reducing psychological disturbances could possibly decelerate the yearly rate of change in FEV1 [22, 33]. Our results highlight the value of periodic spirometric evaluation and the need to conduct intervention studies; maintaining optimal pulmonary health might prevent cardiovascular risk in later life.

### Limitations

However, a few limitations of this study should be acknowledged. Firstly, although both multivariable models and a serial of sensitivity analyses were performed, the effects of unmeasured cofounders could not be eliminated. Secondly, since the majority of the study participants were white US people, the results could not be generalized to more heterogeneous populations. In addition, owing to the observational nature of this study, the reverse causation of lung function decline and an occult illness could not be distinguished. However, the consistency, temporality, and biological gradient of our results could to some extent provide some evidence.

## Conclusions

From this study, we observed a monotonic increase in risks of cardiovascular events with a faster decline in FEV1 and FVC. The inverse association was generally consistent in male or female, white or non-white, smokers or non-smokers, and normal weight or obese populations, but more evident in younger adults. These findings emphasize the value of periodic evaluation of lung function in the general population. Although maintaining optimal pulmonary health could be a potential strategy for preventing cardiovascular events in later life, further intervention studies are warranted.

## Supplementary Information

**Additional file 1.** Detail description of the included cohorts.

**Additional file 2: Table S1.** Baseline characteristics of the study population by quartiles of FEV1decline.

**Additional file 3: Table S2.** Baseline characteristics of the study population by quartiles of FVC decline.

**Additional file 4: Table S3.** Baseline characteristics of participants according to different cohorts.

**Additional file 5: Table S4.** Hazard ratios (95%CIs) of the secondary outcomes with quartiles of FEV1 decline stratified by predefined subgroups.

**Additional file 6: Table S5.** Hazard ratios (95%CIs) of the secondary outcomes with quartiles of FVC decline stratified by predefined subgroups.

**Additional file 7: Table S6.** Hazard ratios (95%CIs) of the studied outcomes with quartiles of FEV1 or FVC decline excluding participants with missing-values on baseline covariates (*n* = 11,067).

**Additional file 8: Table S7.** Hazard ratios (95%CIs) of the studied outcomes with quartiles of FEV1 or FVC decline restricted to participants with no known history of CHD, CHF and COPD at baseline (*n* = 10821).

## Data Availability

The datasets used and/or analyzed during the current study are available from the BioLINCC website on reasonable application.

## Abbreviations

FEV1: Forced respiratory volume in 1 s
FVC: Forced vital capacity
