# Supplementary material for: Longitudinal change in lung function and subsequent risks of cardiovascular events: evidence from four prospective cohort studies
Source: BMC Med. 2021 Jul 2;19:153. doi: 10.1186/s12916-021-02023-3 (PMC8252272; doi:10.1186/s12916-021-02023-3)
Supplement: Supplementary file 1 — Additional file 1. Detail description of the included cohorts. [file 12916_2021_2023_MOESM1_ESM.docx]

**Additional file 1: Detail description of the included cohorts.**

1. *The Coronary Artery Risk Development in Young Adults (CARDIA)*

CARDIA is a prospective cohort study of the evolution of cardiovascular risk factors in young adults (1). Briefly, from 1985 to 1986, 5115 black and white individuals aged 18 to 30 years were examined in Birmingham, AL; Chicago, IL; Minneapolis, MN; and Oakland, CA. In-person examinations occurred at 2, 5, 7, 10, 15, 20, 25, and 30 years. The study was approved by the institutional review boards of each CARDIA field center and written informed consent was obtained from all participants at each examination.

Lung function was measured at baseline and at the year 2, year 5, year 10, and year 20 examinations. Standard spirometry procedures as recommended by the American Thoracic Society were followed at each examination in which lung function was measured. Lung function measurements including FVC (the volume of air that can be forcibly exhaled from the lungs after taking the deepest breath possible) and FEV1 (the volume of air exhaled in the first second of a forced exhalation maneuver).

Participants were contacted annually to inquire about interim hospitalizations or outpatient vascular procedures. Vital status was assessed at 6-month intervals. For each event, medical records were obtained and adjudicated by 2 physician members of the adjudication committee. Disagreements were resolved by full committee review.

1. *The Cardiovascular Health Study (CHS)*

CHS is a population-based epidemiological study designed to investigate cardiovascular disease in the elderly (2). Briefly, 5201 men and women aged 65 or older were initially recruited in 1989–1990 from Medicare eligibility lists in 4 US communities: Forsyth County, North Carolina; Sacramento County, California; Washington County, Maryland; and Pitts- burgh, Pennsylvania. An additional 687 black participants were recruited in a similar fashion between 1992 and 1993. All participants gave written informed consent and the institutional review board at each study site approved the protocol.

Spirometric tests were conducted using a volume displacement water sealed spirometer. At least three acceptable spirograms were obtained from a minimum of five forced expirations. The best single spirogram was identified by computer and confirmed by a technician. Quality assurance was provided by the CHS Pulmonary Function Center and the procedures followed contemporary American Thoracic Society guidelines. Several measures of lung function were used: FEV1 and FVC. The follow-up lung function measurements were conducted at visit 6 (approximately 4 years from baseline visit).

Participants were followed for adjudicated heart failure hospitalization, cardiovascular mortality, and all-cause mortality from year 1994–1995 until the date of outcome of interest, loss to follow-up, or the end of the follow-up period on June 30, 2009. Data on all events of interest were gathered from annual examinations, as well as interim 6-month phone contacts, and confirmed by independent review committee using information from hospital records, death certificates, autopsy reports, and interviews with physicians or next of kin.

1. *The Framingham Heart Study (FHS)*

FHS is a longitudinal community-based cohort study that began in 1948(3). Briefly, 5209 men and women aged 30-62 years at entry were examined biennially for the development of cardiovascular disease. Descriptions of the sampling procedure, methods used in the study, and analytical techniques employed have also been noted in detail elsewhere.

FVC measurements were routinely made at each biennial examination using Collins Respirometer on all but two examinations. FEV1 was also measured at the time of the fifth and tenth biennial examinations. The best of three efforts was used.

Participants have been undergoing routine surveillance for incident cardiovascular events since the baseline examination in 1987 to 1990. The cardiovascular events and causes of death were assigned by a panel of investigators using all the available information from routine biennial clinic examinations, hospital protocols, death certificate information, medical examiners’ reports, autopsies, and information provided by the family and attending physician.

1. *The Framingham Offspring cohort (FHS-OS)*

FHS-OS was started between 1971 and 1975 and includes 5,124 males and females. Participants are the adult children, and their spouses, of the participants in the original Framingham cohort, a seminal study that started in 1948 (4). The Framingham Offspring participants underwent periodic clinical examinations every 4 years up to exam 7, conducted in 1998. The protocol for the FHS-OS was approved by the Boston University Medical Center Institutional Review Board, and all participants provided written informed consent.

Spirometry was performed using a 6L Collins water-sealed bell spirometer connected to an Eagle II microprocessor that provided automatic correction for body temperature, pressure saturated with water vapor conditions, based on calibrations performed daily by technicians. Measurements were obtained with the subject standing and wearing nose clips. Maneuvers were repeated (up to a maximum of 8) until at least three acceptable spirograms were obtained. The largest FVC and the largest FEV1 from all acceptable maneuvers were selected. In the analysis presented here, we include data obtained in exam 1 (1971– 1975), exam 2 (1979–1982), exam 5 (1991–1995), all of which had reliable spirometric measurements and relevant clinical information according to internal National Heart, Lung, and Blood Institute–National Institutes of Health standards.

Participants are under continuous surveillance for cardiovascular events and death. An endpoint committee of three senior investigators reviews all available information including hospital records, death certificates, and next-of-kin interviews to determine the date and cause of death. Cardiovascular events (coronary heart disease: including coronary insufficiency, myocardial infarction, coronary heart disease death; stroke, heart failure, and coronary or cardiovascular disease death) are adjudicated by the committee using standardized criteria.

Reference

1. Friedman GD, Cutter GR, Donahue RP, Hughes GH, Hulley SB, Jacobs DR, et al. Cardia: study design, recruitment, and some characteristics of the examined subjects. Journal of Clinical Epidemiology. 1988;41(11):1105-16.

2. Fried LP, Borhani NO, Enright P, Furberg CD, Gardin JM, Kronmal RA, et al. The cardiovascular health study: Design and rationale. Annals of Epidemiology. 1991;1(3):263-76.

3. Kannel WB, Hubert H, Lew EA. Vital capacity as a predictor of cardiovascular disease: The Framingham study. American Heart Journal. 1983;105(2):311-5.

4. An Investigation of Coronary Heart Disease in Families: The Framingham Offspring Study. American Journal of Epidemiology. 2017;185(11):1093-102.
