# Supplementary material for: Longitudinal change in lung function and subsequent risks of cardiovascular events: evidence from four prospective cohort studies
Source: BMC Med. 2021 Jul 2;19:153. doi: 10.1186/s12916-021-02023-3 (PMC8252272; doi:10.1186/s12916-021-02023-3)
Supplement: Supplementary file 2 — Additional file 2: Table S1. Baseline characteristics of the study population by quartiles of FEV1decline. [file 12916_2021_2023_MOESM2_ESM.docx]

Additional file 2: Table 1 Baseline characteristics of the study population by quartiles of FEV1decline.

|  | Total | Q1 | Q2 | Q3 | Q4 | P value |
| --- | --- | --- | --- | --- | --- | --- |
| No. of participants | 12899 | 3224 | 3225 | 3225 | 3225 |  |
| Age, years | 48.58(21.15) | 48.84(21.33) | 54.61(18.91) | 50.65(19.93) | 40.25(21.68) | <0.001 |
| Sex, male | 5625(43.61%) | 1726(53.54%) | 1315(40.78%) | 1232(38.20%) | 1352(41.9%) | <0.001 |
| Race |  |  |  |  |  | <0.001 |
| Non-white | 2799(21.70%) | 772(23.95%) | 390(12.09%) | 461(14.29%) | 1176(36.47%) |  |
| White | 10100(78.30%) | 2452(76.05%) | 2835(87.91%) | 2764(85.71%) | 2049(63.53%) |  |
| Baseline BMI, kg/m^2^ | 25.60(4.69) | 25.85(4.68) | 25.70(4.46) | 25.58(4.62) | 25.28(4.95) | <0.001 |
| Education level |  |  |  |  |  |  |
| Less than high school | 1355(10.82%) | 354(11.23%) | 394(12.72%) | 337(10.92%) | 270(8.47%) | <0.001 |
| High school graduate | 5594(44.68%) | 1446(45.88%) | 1422(45.92%) | 1332(43.18%) | 1394(43.74%) |  |
| Some college or college+ | 5572(44.50%) | 1352(42.89%) | 1281(41.36%) | 1416(45.90%) | 1523(47.79%) |  |
| Marital status |  |  |  |  |  | <0.001 |
| Married | 6293(50.26%) | 1375(43.62%) | 1846(59.61%) | 1835(59.48%) | 1237(38.81%) |  |
| Separated/divorced/widowed | 1340(10.70%) | 286(9.07%) | 445(14.37%) | 381(12.35%) | 228(7.15%) |  |
| Never married | 4888(39.04%) | 1491(47.30%) | 806(26.03%) | 869(28.17%) | 1722(54.03%) |  |
| Smoking status |  |  |  |  |  | <0.001 |
| Never | 6172(47.85%) | 1429(44.32%) | 1513(46.91%) | 1547(47.97%) | 1683(52.19%) |  |
| Former | 2932(22.73%) | 660(20.47%) | 833(25.83%) | 847(26.26%) | 592(18.36%) |  |
| Current | 3795(29.42%) | 1135(35.20%) | 879(27.26%) | 831(25.77%) | 950(29.46%) |  |
| Current alcohol use |  |  |  |  |  | 0.30 |
| No | 4875(37.79%) | 1201(37.25%) | 1192(36.96%) | 1221(37.86%) | 1261(37.10%) |  |
| Yes | 8024(62.21%) | 2023(62.75%) | 2033(63.04%) | 2004(62.14%) | 1964(60.90%) |  |
| Physical activity, MET-min/week | 1767(1895) | 1784(1848) | 1834(2153) | 1816(2023) | 1631(1469) | <0.001 |
| History |  |  |  |  |  |  |
| Diabetes | 583(4.52%) | 123(3.82%) | 160(4.96%) | 163(5.05%) | 137(4.25%) | 0.05 |
| Hypertension | 2282(17.69%) | 540(16.75%) | 621(19.26%) | 603(18.71%) | 518(16.06%) | 0.001 |
| CHD | 891(6.91%) | 215(6.67%) | 236(7.32%) | 215(6.67%) | 225(6.98%) | 0.7 |
| CHF | 695(5.39%) | 160(4.96%) | 179(5.55%) | 183(5.67%) | 173(5.36%) | 0.61 |
| COPD | 492(3.83%) | 96(3.00%) | 157(4.88%) | 149(4.63%) | 90(2.80%) | <0.001 |
| Glucose, mmol/l | 5.23(1.34) | 5.11(1.34) | 5.41(1.27) | 5.41(1.34) | 4.99(1.37) | 0.001 |
| Total cholesterol, mg/dL | 5.04(1.03) | 5.03(1.01) | 5.19(1.04) | 5.07(1.04) | 4.89(1.02) | 0.25 |
| HDL cholesterol, mg/dL | 1.37(0.38) | 1.34(0.37) | 1.39(0.39) | 1.39(0.38) | 1.38(0.36) | <0.001 |
| LDL cholesterol, mg/dL | 3.16(0.92) | 3.20(0.93) | 3.26(0.91) | 3.15(0.93) | 3.03(0.91) | 0.36 |
| Triglyceride, mg/dL | 1.67(1.67) | 1.61(1.69) | 1.93(1.83) | 1.87(1.75) | 1.27(1.25) | <0.001 |
| Annual change of FEV1, Liters/year | -0.022(0.094) | -0.101(0.093) | -0.037(0.007) | -0.015(0.007) | 0.065(0.111) | <0.001 |
| Annual change of FVC, Liters/year | -0.021(0.096) | -0.079(0.094) | -0.038(0.045) | -0.015(0.043) | 0.049(0.124) | <0.001 |

Abbreviation: BMI=body mass index; CHD=coronary heart disease; CHF=chronic heart failure; COPD=chronic obstructive pulmonary disease; HDL=high-density lipoprotein; LDL=low-density lipoprotein; FEV1=forced expiratory volume in one second; FEV1= forced expiratory volume in one second.
