# Supplementary material for: Longitudinal change in lung function and subsequent risks of cardiovascular events: evidence from four prospective cohort studies
Source: BMC Med. 2021 Jul 2;19:153. doi: 10.1186/s12916-021-02023-3 (PMC8252272; doi:10.1186/s12916-021-02023-3)
Supplement: Supplementary file 3 — Additional file 3: Table S2. Baseline characteristics of the study population by quartiles of FVC decline. [file 12916_2021_2023_MOESM3_ESM.docx]

Additional file 3: Table 2 Baseline characteristics of the study population by quartiles of FVC decline.

|  | Total | Q1 | Q2 | Q3 | Q4 | P value |
| --- | --- | --- | --- | --- | --- | --- |
| No. of participants | 12899 | 3224 | 3225 | 3225 | 3225 |  |
| Age, years | 48.58(21.15) | 56.06(20.66) | 53.78(19.29) | 45.89(19.08) | 38.62(20.92) | <0.001 |
| Sex, male | 5625(43.61%) | 1713(53.13%) | 1233(38.23%) | 1268(39.32%) | 1411(43.74%) | <0.001 |
| Race |  |  |  |  |  | <0.001 |
| Non-white | 2799(21.70%) | 746(23.14%) | 451(13.98%) | 551(17.09%) | 1051(32.59%) |  |
| White | 10100(78.30%) | 2478(76.86%) | 2774(86.02%) | 2674(82.91%) | 2174(67.41%) |  |
| Baseline BMI, kg/m^2^ | 25.60(4.69) | 26.21(4.40) | 25.97(4.58) | 25.47(4.88) | 24.77(4.74) | <0.001 |
| Education level |  |  |  |  |  | <0.001 |
| Less than high school | 1355(10.82%) | 484(15.26%) | 383(12.35%) | 260(8.51%) | 228(7.14%) |  |
| High school graduate | 5594(44.68%) | 1514(47.75%) | 1412(45.53%) | 1266(41.44%) | 1402(43.89%) |  |
| Some college or college+ | 5572(44.50%) | 1173(36.99%) | 1306(42.12%) | 1529(50.05%) | 1564(48.97%) |  |
| Marital status |  |  |  |  |  | <0.001 |
| Married | 6293(50.26%) | 1686(53.17%) | 1840(59.34%) | 1667(54.57%) | 1100(34.44%) |  |
| Separated/divorced/widowed | 1340(10.70%) | 477(15.04%) | 442(14.25%) | 267(8.74%) | 154(4.82%) |  |
| Never married | 4888(39.04%) | 1008(31.79%) | 819(26.41%) | 1121(36.69%) | 1940(60.74%) |  |
| Smoking status |  |  |  |  |  | <0.001 |
| Never | 6172(47.85%) | 1402(43.49%) | 1571(48.71%) | 1534(47.57%) | 1665(51.63%) |  |
| Former | 2932(22.73%) | 956(29.65%) | 810(25.12%) | 690(21.40%) | 476(14.76%) |  |
| Current | 3795(29.42%) | 866(26.86%) | 844(26.17%) | 1001(31.04%) | 1084(33.61%) |  |
| Current alcohol use |  |  |  |  |  | 0.06 |
| No | 4875(37.79%) | 1266(39.27%) | 1185(36.74%) | 1179(36.56%) | 1245(38.60%) |  |
| Yes | 8024(62.21%) | 1958(60.73%) | 2040(63.26%) | 2046(63.44%) | 1980(61.40%) |  |
| Physical activity, MET-min/week | 1767(1895) | 1898(2179) | 1799(2194) | 1718(1690) | 1658(1397) | <0.001 |
| History |  |  |  |  |  |  |
| Diabetes | 583(4.52%) | 204(6.33%) | 182(5.64%) | 111(3.44%) | 86(2.67%) | <0.001 |
| Hypertension | 2282(17.69%) | 811(25.16%) | 642(19.91%) | 445(13.80%) | 384(11.91%) | <0.001 |
| CHD | 891(6.91%) | 320(9.93%) | 235(7.29%) | 145(4.50%) | 191(5.92%) | <0.001 |
| CHF | 695(5.39%) | 137(4.25%) | 172(5.33%) | 205(6.36%) | 181(5.61%) | 0.002 |
| COPD | 492(3.83%) | 211(6.57%) | 145(4.51%) | 69(2.15%) | 67(2.09%) | <0.001 |
| Glucose, mmol/l | 5.23(1.34) | 5.41(1.52) | 5.44(1.30) | 5.21(1.23) | 4.85(1.22) | <0.001 |
| Total cholesterol, mg/dL | 5.04(1.03) | 5.16(1.01) | 5.18(1.03) | 4.99(1.03) | 4.84(1.02) | 0.66 |
| HDL cholesterol, mg/dL | 1.37(0.38) | 1.36(0.39) | 1.39(0.38) | 1.36(0.37) | 1.37(0.36) | <0.001 |
| LDL cholesterol, mg/dL | 3.16(0.92) | 3.23(0.90) | 3.25(0.93) | 3.13(0.91) | 3.02(0.93) | 0.14 |
| Triglyceride, mg/dL | 1.67(1.67) | 1.55(1.31) | 1.87(1.73) | 1.99(2.06) | 1.28(1.37) | <0.001 |
| Annual change of FEV1, Liters/year | -0.022(0.094) | -0.072(0.098) | -0.032(0.050) | -0.018(0.060) | 0.033(0.118) | <0.001 |
| Annual change of FVC, Liters/year | -0.021(0.096) | -0.113(0.076) | -0.040(0.010) | -0.007(0.010) | 0.076(0.111) | <0.001 |

Abbreviation: BMI=body mass index; CHD=coronary heart disease; CHF=chronic heart failure; COPD=chronic obstructive pulmonary disease; HDL=high-density lipoprotein; LDL=low-density lipoprotein; FEV1=forced expiratory volume in one second; FVC=forced vital capacity.
