# Supplementary material for: Longitudinal change in lung function and subsequent risks of cardiovascular events: evidence from four prospective cohort studies
Source: BMC Med. 2021 Jul 2;19:153. doi: 10.1186/s12916-021-02023-3 (PMC8252272; doi:10.1186/s12916-021-02023-3)
Supplement: Supplementary file 4 — Additional file 4: Table S3. Baseline characteristics of participants according to different cohorts. [file 12916_2021_2023_MOESM4_ESM.docx]

Additional file 4: Table 3 Baseline characteristics of participants according to different cohorts.

|  | Total | CARDIA | CHS | FHS | FHS-OS |
| --- | --- | --- | --- | --- | --- |
| No. of participants | 12899 | 4715 | 3924 | 1427 | 2833 |
| Age, years | 48.58(21.15) | 24.83(3.61) | 71.33(3.90) | 68.91(6.37) | 46.54(9.69) |
| Sex, male | 5625(43.61%) | 2154(45.68%) | 1625(41.41%) | 537(37.63%) | 1309(46.21%) |
| Race |  |  |  |  |  |
| Non-white | 2799(21.70%) | 2349(49.82%) | 450(11.47%) | 0 | 0 |
| White | 10100(78.30%) | 2366(50.18%) | 3474(88.53%) | 1427(100%) | 2833(100%) |
| Baseline BMI, kg/m^2^ | 25.60(4.69) | 24.46(4.79) | 26.56(3.98) | 26.51(4.18) | 25.74(5.25) |
| Education level |  |  |  |  |  |
| Less than high school | 1355(10.82%) | 20(0.42%) | 957(24.39%) | 329(23.06%) | 49(2.00%) |
| High school graduate | 5594(44.68%) | 1823(38.66%) | 2084(53.11%) | 792(55.50%) | 895(36.46%) |
| Some college or college+ | 5572(44.50%) | 2872(60.91%) | 883(22.50%) | 306(21.44%) | 1511(61.55%) |
| Marital status |  |  |  |  |  |
| Married | 6293(50.26%) | 1435(30.43%) | 2709(69.04%) | 122(8,55%) | 2027(82.57%) |
| Separated/divorced/widowed | 1340(10.70%) | 0 | 1062(27.06%) | 54(3.78%) | 224(9.12%) |
| Never married | 4888(39.04%) | 3280(69.57%) | 153(3.90%) | 1251(87.67%) | 204(8.31%) |
| Smoking status |  |  |  |  |  |
| Never | 6172(47.85%) | 2678(56.80%) | 1841(46.92%) | 570(39.94%) | 1083(38.23%) |
| Former | 2932(22.73%) | 615(13.04%) | 1655(42.18%) | 85(5.96%) | 577(20.37%) |
| Current | 3795(29.42%) | 1422(30.16%) | 428(10.91%) | 722(54.10%) | 1173(41.40%) |
| Current alcohol use |  |  |  |  |  |
| No | 4875(37.79%) | 1829(38.79%) | 1805(46.00%) | 437(30.62%) | 804(28.38%) |
| Yes | 8024(62.21%) | 2886(61.21%) | 2119(54.00%) | 990(69.38%) | 2029(71.62%) |
| Physical activity, MET-min/week | 1682(1232) | 2073(2661) | 1621(1209) | 1573(1809) | 1767(1895) |
| History |  |  |  |  |  |
| Diabetes | 583(4.52%) | 39(0.83%) | 522(13.30%) | 0 | 22(0.78%) |
| Hypertension | 2282(17.69%) | 427(9.06%) | 1596(40.67%) | 83(5.82%) | 176(6.22%) |
| CHD | 891(6.91%) | 233(4.94%) | 657(16.74%) | 0 | 1(0.04%) |
| CHF | 695(5.39%) | 278(5.90%) | 100(2.55%) | 0 | 317(5.39%) |
| COPD | 492(3.83%) | 0 | 459(11.70%) | 33(2.40%) | 0 |
| Glucose, mmol/l | 5.23(1.34) | 4.59(0.89) | 6.03(1.74) | 4.41(0.72) | 5.60(0.59) |
| Total cholesterol, mg/dL | 5.04(1.03) | 4.58(0.86) | 5.48(1.00) | 5.52(1.05) | 4.96(0.96) |
| HDL cholesterol, mg/dL | 1.37(0.38) | 1.37(0.34) | 1.41(0.40) | 1.27(0.45) | 1.33(0.37) |
| LDL cholesterol, mg/dL | 3.16(0.92) | 2.83(0.81) | 3.37(0.91) | 3.86(0.95) | 3.13(0.86) |
| Triglyceride, mg/dL | 1.67(1.67) | 0.83(0.55) | 1.57(0.81) | 1.50(0.80) | 3.29(2.58) |
| Baseline FEV1, liter | 2.77(0.96) | 3.35(0.85) | 2.15(0.65) | 2.05(0.70) | 3.12(0.78) |
| Baseline FVC, liter | 3.58(1.13) | 4.13(1.05) | 3.05(0.86) | 2.61(0.87) | 4.03(0.96) |

Abbreviation: CARDIA= The Coronary Artery Risk Development in Young Adults; CHS= The Cardiovascular Health Study; FHS= The Framingham Heart Study; FHS-OS= The Framingham Offspring cohort; BMI=body mass index; CHD=coronary heart disease; CHF=chronic heart failure; COPD=chronic obstructive pulmonary disease; HDL=high-density lipoprotein; LDL=low-density lipoprotein; FEV1=forced expiratory volume in one second; FVC=forced vital capacity.
