# Supplementary material for: Longitudinal change in lung function and subsequent risks of cardiovascular events: evidence from four prospective cohort studies
Source: BMC Med. 2021 Jul 2;19:153. doi: 10.1186/s12916-021-02023-3 (PMC8252272; doi:10.1186/s12916-021-02023-3)
Supplement: Supplementary file 5 — Additional file 5: Table S4. Hazard ratios (95%CIs) of the secondary outcomes with quartiles of FEV1 decline stratified by predefined subgroups. [file 12916_2021_2023_MOESM5_ESM.docx]

Additional file 5: Table 4 Hazard ratios (95%CIs) of the secondary outcomes with quartiles of FEV1 decline stratified by predefined subgroups.

|  | Q1 | Q2 | Q3 | Q4 | *P* for interaction |
| --- | --- | --- | --- | --- | --- |
| ***Coronary heart disease*** | |  |  |  |  |
| Age, years |  |  |  |  |  |
| <60 | 1.27(0.96,1.69) | 1.07(0.79,1.45) | 0.84(0.62,1.14) | Reference | 0.04 |
| >=60 | 1.36(1.08,1.71) | 1.00(0.79,1.27) | 1.10(0.86,1.39) | Reference |  |
| Sex |  |  |  |  |  |
| Women | 1.48(1.14,1.92) | 1.05(0.81,1.37) | 1.00(0.77,1.31) | Reference | 0.22 |
| Men | 1.34(1.05,1.71) | 1.11(0.86,1.44) | 1.08(0.83,1.42) | Reference |  |
| Race |  |  |  |  |  |
| Non-white | 2.10(1.32,3.36) | 0.77(0.39,1.51) | 1.56(0.90,2.71) | Reference | 0.38 |
| White | 1.32(1.08,1.60) | 1.08(0.89,1.31) | 1.00(0.82,1.22) | Reference |  |
| Baseline BMI |  |  |  |  |  |
| Normal | 1.28(0.96,1.71) | 0.87(0.64,1.18) | 0.93(0.68,1.27) | Reference | 0.74 |
| Overweight | 1.44(1.07,1.93) | 1.24(0.92,1.68) | 1.13(0.83,1.53) | Reference |  |
| Obese | 1.43(1.00,2.06) | 1.12(0.77,1.63) | 1.09(0.74,1.60) | Reference |  |
| Smoking status |  |  |  |  |  |
| Never | 1.22(0.92,1.62) | 0.94(0.71,1.25) | 0.96(0.72,1.29) | Reference | 0.03 |
| Former | 1.58(1.08,2.31) | 1.36(0.94,1.97) | 1.08(0.74,1.57) | Reference |  |
| Current | 1.46(1.09,1.96) | 1.00(0.72,1.40) | 1.10(0.78,1.54) | Reference |  |
| ***Chronic heart failure*** | |  |  |  |  |
| Age, years |  |  |  |  |  |
| <60 | 1.27(0.87,1.86) | 1.29(0.88,1.91) | 1.00(0.67,1.48) | Reference | 0.04 |
| >=60 | 1.47(1.23,1.76) | 1.37(1.15,1.63) | 1.38(1.15,1.65) | Reference |  |
| Sex |  |  |  |  |  |
| Women | 1.23(0.99,1.53) | 1.28(1.05,1.56) | 1.14(0.93,1.41) | Reference | 0.39 |
| Men | 1.85(1.44,2.38) | 1.57(1.21,2.04) | 1.63(1.26,2.13) | Reference |  |
| Race |  |  |  |  |  |
| Non-white | 1.71(1.14,2.57) | 1.17(0.72,1.88) | 1.17(0.73,1.89) | Reference | 0.14 |
| White | 1.47(1.23,1.75) | 1.43(1.20,1.70) | 1.36(1.14,1.62) | Reference |  |
| Baseline BMI |  |  |  |  |  |
| Normal | 1.68(1.24,2.28) | 1.36(1.00,1.83) | 1.35(0.98,1.85) | Reference | 0.37 |
| Overweight | 1.36(1.05,1.76) | 1.43(1.11,1.84) | 1.34(1.04,1.74) | Reference |  |
| Obese | 1.37(1.03,1.84) | 1.37(1.03,1.82) | 1.34(1.00,1.79) | Reference |  |
| Smoking status |  |  |  |  |  |
| Never | 1.31(1.02,1.67) | 1.20(0.95,1.52) | 1.14(0.89,1.45) | Reference | 0.46 |
| Former | 2.38(1.69,3.37) | 2.13(1.53,2.96) | 1.99(1.43,2.77) | Reference |  |
| Current | 1.18(0.89,1.56) | 1.14(0.84,1.55) | 1.10(0.80,1.53) | Reference |  |
| ***Stroke*** |  |  |  |  |  |
| Age, years |  |  |  |  |  |
| <60 | 1.52(1.05,2.21) | 1.33(0.89,1.97) | 0.88(0.58,1.34) | Reference | 0.003 |
| >=60 | 1.24(0.98,1.57) | 1.29(1.03,1.62) | 1.12(0.88,1.42) | Reference |  |
| Sex |  |  |  |  |  |
| Women | 1.30(1.01,1.67) | 1.22(0.96,1.56) | 0.94(0.72,1.21) | Reference | 0.51 |
| Men | 1.47(1.07,2.03) | 1.56(1.12,2.17) | 1.37(0.96,1.94) | Reference |  |
| Race |  |  |  |  |  |
| Non-white | 1.43(0.90,2.26) | 1.04(0.58,1.89) | 1.41(0.85,2.35) | Reference | 0.14 |
| White | 1.34(1.08,1.67) | 1.38(1.12,1.71) | 1.06(0.85,1.34) | Reference |  |
| Baseline BMI |  |  |  |  |  |
| Normal | 1.40(1.01,1.94) | 1.34(0.97,1.85) | 1.15(0.82,1.62) | Reference | 0.40 |
| Overweight | 1.28(0.94,1.75) | 1.27(0.94,1.73) | 0.97(0.70,1.35) | Reference |  |
| Obese | 1.39(0.92,2.11) | 1.39(0.92,2.11) | 1.22(0.79,1.89) | Reference |  |
| Smoking status |  |  |  |  |  |
| Never | 1.44(1.06,1.96) | 1.33(0.99,1.78) | 1.16(0.85,1.57) | Reference | 0.57 |
| Former | 1.34(0.86,2.08) | 1.37(0.90,2.08) | 1.08(0.71,1.66) | Reference |  |
| Current | 1.27(0.92,1.75) | 1.38(0.98,1.95) | 0.95(0.64,1.40) | Reference |  |

Adjusted model: adjusted for age, sex, race, education level, marital status, history of hypertension, diabetes, coronary heart disease, heart failure, chronic obstructive pulmonary disease, smoking status, current alcoholic use, physical activity, body mass index, fasting serum glucose, total cholesterol, high-density lipoprotein cholesterol, triglycerides and low-density lipoprotein cholesterol. FEV1=forced expiratory volume in one second.
