# Supplementary material for: Longitudinal change in lung function and subsequent risks of cardiovascular events: evidence from four prospective cohort studies
Source: BMC Med. 2021 Jul 2;19:153. doi: 10.1186/s12916-021-02023-3 (PMC8252272; doi:10.1186/s12916-021-02023-3)
Supplement: Supplementary file 7 — Additional file 7: Table S6. Hazard ratios (95%CIs) of the studied outcomes with quartiles of FEV1 or FVC decline excluding participants with missing-values on baseline covariates (n = 11,067). [file 12916_2021_2023_MOESM7_ESM.docx]

Additional file 7: Table 6 Hazard ratios (95%CIs) of the studied outcomes with quartiles of FEV1 or FVC decline excluding participants with missing-values on baseline covariates (n=11,067).

| Studied outcomes | Model | Q1 | Q2 | Q3 | Q4 |
| --- | --- | --- | --- | --- | --- |
| ***FEV1 decline*** |  |  |  |  |  |
| Cardiovascular events | Non-adjusted | 1.95(1.74,2.19) | 2.42(2.16,2.71) | 1.67(1.48,1.88) | Reference |
|  | Adjusted | 1.40(1.21,1.62) | 1.26(1.10,1.46) | 1.06(0.91,1.22) | Reference |
| Coronary heart disease | Non-adjusted | 2.05(1.72,2.44) | 2.05(1.71,2.45) | 1.67(1.39,2.00) | Reference |
|  | Adjusted | 1.34(1.08,1.67) | 1.05(0.85,1.30) | 0.97(0.78,1.21) | Reference |
| Chronic heart failure | Non-adjusted | 2.02(1.72,2.36) | 2.53(2.17,2.96) | 1.98(1.69,2.33) | Reference |
|  | Adjusted | 1.54(1.26,1.89) | 1.38(1.14,1.67) | 1.31(1.08,1.59) | Reference |
| Stroke | Non-adjusted | 1.94(1.60,2.36) | 2.49(2.06,3.01) | 1.65(1.35,2.02) | Reference |
|  | Adjusted | 1.68(1.31,2.16) | 1.35(1.05,1.72) | 1.11(0.86,1.43) | Reference |
| ***FVC decline*** |  |  |  |  |  |
| Cardiovascular events | Non-adjusted | 2.72(2.43,3.04) | 2.21(1.97,2.47) | 1.47(1.31,1.66) | Reference |
|  | Adjusted | 1.31(1.13,1.52) | 1.16(1.00,1.35) | 1.07(0.92,1.25) | Reference |
| Coronary heart disease | Non-adjusted | 2.89(2.41,3.46) | 2.42(2.01,2.90) | 1.74(1.44,2.10) | Reference |
|  | Adjusted | 1.57(1.24,2.00) | 1.42(1.12,1.80) | 1.31(1.03,1.67) | Reference |
| Chronic heart failure | Non-adjusted | 3.22(2.77,3.75) | 2.45(2.10,2.86) | 1.43(1.21,1.69) | Reference |
|  | Adjusted | 1.24(1.01,1.51) | 1.07(0.87,1.30) | 0.90(0.73,1.12) | Reference |
| Stroke | Non-adjusted | 2.67(2.22,3.22) | 2.23(1.85,2.69) | 1.25(1.02,1.53) | Reference |
|  | Adjusted | 1.58(1.22,2.05) | 1.28(0.99,1.66) | 1.08(0.82,1.42) | Reference |

Adjusted model: adjusted for age, sex, race, education level, marital status, history of hypertension, diabetes, coronary heart disease, heart failure, chronic obstructive pulmonary disease, smoking status, current alcoholic use, physical activity, body mass index, fasting serum glucose, total cholesterol, high-density lipoprotein cholesterol, triglycerides and low-density lipoprotein cholesterol. FEV1=forced expiratory volume in one second; FVC=forced vital capacity.
