# Supplementary material for: Longitudinal change in lung function and subsequent risks of cardiovascular events: evidence from four prospective cohort studies
Source: BMC Med. 2021 Jul 2;19:153. doi: 10.1186/s12916-021-02023-3 (PMC8252272; doi:10.1186/s12916-021-02023-3)
Supplement: Supplementary file 8 — Additional file 8: Table S7. Hazard ratios (95%CIs) of the studied outcomes with quartiles of FEV1 or FVC decline restricted to participants with no known history of CHD, CHF and COPD at baseline (n = 10821). [file 12916_2021_2023_MOESM8_ESM.docx]

Additional file 8: Table 7 Hazard ratios (95%CIs) of the studied outcomes with quartiles of FEV1 or FVC decline restricted to participants with no known history of CHD, CHF and COPD at baseline (n=10821).

| Studied outcomes | Model | Q1 | Q2 | Q3 | Q4 |
| --- | --- | --- | --- | --- | --- |
| ***FEV1 decline*** |  |  |  |  |  |
| Cardiovascular events | Non-adjusted | 2.02(1.79,2.29) | 2.45(2.16,2.76) | 1.63(1.43,1.86) | Reference |
|  | Adjusted | 1.31(1.16,1.49) | 1.26(1.11,1.43) | 1.03(0.90,1.18) | Reference |
| Coronary heart disease | Non-adjusted | 2.01(1.66,2.44) | 2.06(1.70,2.51) | 1.60(1.30,1.96) | Reference |
|  | Adjusted | 1.30(1.07,1.58) | 1.04(0.85,1.27) | 0.99(0.80,1.22) | Reference |
| Chronic heart failure | Non-adjusted | 2.07(1.74,2.47) | 2.53(2.13,3.01) | 1.91(1.60,2.29) | Reference |
|  | Adjusted | 1.44(1.20,1.71) | 1.31(1.10,1.57) | 1.27(1.06,1.53) | Reference |
| Stroke | Non-adjusted | 2.05(1.66,2.54) | 2.66(2.16,3.28) | 1.65(1.32,2.06) | Reference |
|  | Adjusted | 1.40(1.13,1.73) | 1.40(1.13,1.73) | 1.08(0.86,1.36) | Reference |
| ***FVC decline*** |  |  |  |  |  |
| Cardiovascular events | Non-adjusted | 2.70(2.39,3.05) | 2.23(1.97,2.53) | 1.51(1.33,1.72) | Reference |
|  | Adjusted | 1.35(1.19,1.53) | 1.21(1.07,1.38) | 1.11(0.98,1.27) | Reference |
| Coronary heart disease | Non-adjusted | 2.71(2.23,3.30) | 2.33(1.91,2.85) | 1.72(1.40,2.10) | Reference |
|  | Adjusted | 1.43(1.16,1.76) | 1.31(1.07,1.61) | 1.24(1.00,1.52) | Reference |
| Chronic heart failure | Non-adjusted | 3.19(2.70,3.78) | 2.45(2.06,2.91) | 1.44(1.20,1.73) | Reference |
|  | Adjusted | 1.44(1.21,1.71) | 1.26(1.06,1.51) | 1.08(0.90,1.30) | Reference |
| Stroke | Non-adjusted | 2.77(2.25,3.39) | 2.39(1.95,2.94) | 1.33(1.06,1.66) | Reference |
|  | Adjusted | 1.40(1.13,1.73) | 1.33(1.07,1.64) | 1.02(0.81,1.28) | Reference |

Adjusted model: adjusted for age, sex, race, education level, marital status, history of hypertension, diabetes, coronary heart disease, heart failure, chronic obstructive pulmonary disease, smoking status, current alcoholic use, physical activity, body mass index, fasting serum glucose, total cholesterol, high-density lipoprotein cholesterol, triglycerides and low-density lipoprotein cholesterol. FEV1=forced expiratory volume in one second; FVC=forced vital capacity; CHD=coronary heart disease; CHF=chronic heart failure; COPD=chronic obstructive pulmonary disease.
